# Using Age Structure for a Multi-stage Optimal Control Model with Random Switching Time

**DOI:** 10.1007/s10957-019-01598-5

**Published:** 2019-12-07

**Authors:** Stefan Wrzaczek, Michael Kuhn, Ivan Frankovic

**Affiliations:** Wittgenstein Centre (IIASA, VID/ÖAW, WU), Vienna Institute of Demography, Vienna, Austria

**Keywords:** Optimal control theory, Age-structured optimal control theory, Multi-stage, Random switch, Catastrophic disaster, 34K35, 49J55, 49K15

## Abstract

The paper presents a transformation of a multi-stage optimal control model with random switching time to an age-structured optimal control model. Following the mathematical transformation, the advantages of the present approach, as compared to a standard backward approach, are discussed. They relate in particular to a compact and unified representation of the two stages of the model: the applicability of well-known numerical solution methods and the illustration of state and control dynamics. The paper closes with a simple example on a macroeconomic shock, illustrating the workings and advantages of the approach.

## Introduction

Optimal control models with a variable time horizon continue to be the object of intensive research interest from both a theoretical and an applied point of view. Contributions can, in principle, be subdivided into two classes: (i) optimal control models with random time horizon and (ii) multi-stage optimal control models.

Class (i) comprises optimal control models that are deterministic in their state variables but stochastic in the time horizon. The decision maker is assumed to know the distribution of the terminal time (which is a random variable) and can thus derive the expected objective function. Once the random variable is realized, the optimal control model terminates and the decision maker obtains some salvage value, possibly depending on the final state and the terminal time. Class (ii) comprises optimal control models with a change in the dynamics and/or in the objective function at a certain switching time. In this stream of the literature, the deterministic switching time is endogenously determined by the decision maker.

While both model classes have been developed and applied extensively (see literature review in [1]), there are but a few examples, where they have been combined (see, e.g., [2–6]), although this seems a necessity, when analyzing settings in which a random transition induces drastic changes in the objective function or the constraints of an optimal control problem (examples given further on below). One reason is that, while such models can be formulated as optimal control models with a random time horizon, they are difficult to solve. For many applications, even a numerical treatment is computationally involved to the point of intractability, as the solution up to the switching time includes an explicit expression of the post-switching value function in terms of the state variables and time.

In this contribution, we consider a general model that changes the dynamics and/or the objective function at a random switching time, characterized by a known distribution depending on the state and the control variables. This implies that the model belongs both to class (i) because of the random termination of the first stage, and to class (ii) because of the assumed change in the dynamics and/or objective in the second stage. We then propose a transformation to a deterministic age-structured optimal control model that allows one to arrive at a convenient and complete presentation of the solution to the original problem. Specifically, the reformulation has the following advantages (for a deeper discussion, we refer to the end of Sect. 2):

1. *Numerical solution* Considering the model as an age-structured optimal control model, a complete numerical solution can be found with well-established methods (see, e.g., [7]).
2. *Analytical insights* If the model is treated as an optimal control model with random time horizon, the solution only describes the stage before the switch. All information concerning stage 2 is implicitly included in the post-switch value function. By treating both stages simultaneously, the new approach allows one to represent the model and its solution in a unified form that expresses explicitly the links between the two stages, and to characterize in a convenient and intuitive way the mechanisms behind the optimal dynamics of the controls and states.

The idea of this reformulation has been briefly suggested in [8] (section 3.5, p. 232), but has not been presented in a formal and exhaustive way. As part of this contribution, we develop the advantages of this method as compared to the classical formulation as an optimal control model with a random time horizon.

Applications of such models are plentiful. In “Appendix,” we sketch three types of setting relating to innovation, to natural disaster and climate change, and to political shocks. Further applications include the analysis of shock-like (health) events over the individual life-cycle (see [6] on the random transition into addiction) and security crises due to, e.g., terror attacks.

The following provides a brief overview of the literature that forms the foundation of our approach. The literature on optimal control models with a stochastic terminal time started with the seminal papers by Yaari [9] (life-cycle model) and Kamien and Schwartz [10] (machine replacement and maintenance model). The theoretical basis for optimal control models with random stopping time has been provided in [11–14]. In these papers, it is shown that the stochastic optimal control problems can be reformulated as deterministic optimal control problems with infinite time horizon. This approach is the starting point of our paper (see Sect. 2).

In multi-stage optimal control models, the time horizon consists of two (or more) stages with different model dynamics and/or objective functions. The switching time is a decision variable, possibly subject to switching costs. The theoretical basis for this literature has been provided in [15–17]. We present a transformation of a multi-stage optimal control model with a random switching time to an age-structured optimal control model. Early models of the latter class dealt with optimal harvesting from age-structured populations (e.g., [18–20]). The Maximum Principles in these papers, however, were specific to the problems. A general version of the Maximum Principle for age-structured optimal control models was first provided by Brokate [21], with [8, 22–24] adding further generalizations.

The remainder of the paper is structured as follows. Section 2 presents the model and its transformation, first to a deterministic optimal control model and subsequently to a deterministic age-structured optimal control model. Section 3 illustrates the method by way of an application to an example relating to the prevention of catastrophic macroeconomic disasters. Conclusions are given in Sect. 4.

## Model Setting and Transformation

In this section, we first present the model and the deterministic representation derived in [11] and then continue with the transformation to an optimal control model with age structure.

### The Model and its Reformulation as a Deterministic Optimal Control Model

Let us assume that the time horizon is separated by the switching time $\tau$ into two stages, subsequently referred to as stages 1 and 2. Here, $\tau$ is a random variable out of the sample space $\Omega = [0, \infty [$. The probability space is then denoted by $(\Omega , \Sigma , {\mathbb {P}})$, with $\Sigma$ denoting the Borel $\sigma$-Algebra on $\Omega$, and $\mathcal {F} (t)$ (with corresponding density $\mathcal {F}' (t)$) denoting the cumulative probability that the model has switched by time *t*, i.e., $\mathcal {F} (t)= \mathbb {P} ( \tau \le t)$. The switching rate, which is assumed to depend continuously on the state and control variables, can then be defined as

1 $$\begin{aligned} \eta (x(t),u(t),t)= \frac{\mathcal {F}' (t)}{1- \mathcal {F} (t)}, \end{aligned}$$

where $\eta : \mathbb {R}^{n} \times \mathbb {R}^{m} \times \mathbb {R} \rightarrow \mathbb {R}$ is a continuous function in the state variable $x(t)\in \mathbb {R}^{n}$, the control variable $u(t)\in \mathbb {R}^{m}$ and *t*.

The dynamics of the model (separated into stages 1 and 2 by the random variable $\tau$) is defined by the following system of ordinary differential equations

2 $$\begin{aligned} \dot{x} (t):= \frac{\mathrm{d}x(t)}{\mathrm{d}t}= & {} \left\{ \begin{array}{ll} f_{1}(x(t),u(t),t) &{} \text {for }t< \tau , \\ f_{2}(x(t),u(t),t,x( \tau ), \tau ) &{} \text {for }t \ge \tau , \end{array} \right. \nonumber \\&x(t_{0})=x_{t_{0}},\qquad x( \tau )= \lim _{t \nearrow \tau } \varphi (x(t),t). \end{aligned}$$

Here, $f_{1}:\mathbb {R}^{n}\times \mathbb {R}^{m}\times \mathbb {R}\rightarrow \mathbb {R}^{n}$ and $f_{2}:\mathbb {R}^{n}\times \mathbb {R}^{m}\times \mathbb {R} \times \mathbb {R}^{n} \times \mathbb {R} \rightarrow \mathbb {R}^{n}$ are assumed to be piecewise continuous in *x*, *u* and *t*; and $\varphi :\mathbb {R}^{n}\times \mathbb {R}\rightarrow \mathbb {R}^{n}$ is assumed to be piecewise continuous in *x* and *t*. We understand $(u(\cdot ),x(\cdot ))$ to be admissible if the measurable control function $u(\cdot )$ and the absolutely continuous state function $x(\cdot )$ solve the dynamic system (2) uniquely.

Let $g_{1}: \mathbb {R}^{n} \times \mathbb {R}^{m} \times \mathbb {R} \rightarrow \mathbb {R}$ and $g_{2}: \mathbb {R}^{n} \times \mathbb {R}^{m} \times \mathbb {R} \times \mathbb {R}^{m} \times \mathbb {R} \rightarrow \mathbb {R}$ be continuous in *x*, *u* and *t* with continuous $\partial g_{i}( \cdot ) / \partial x$. Then, the objective functional is defined by

3 $$\begin{aligned} g(x(t),u(t),t)= \left\{ \begin{array}{ll} g_{1}(x(t),u(t),t) &{} \text {for } t< \tau , \\ g_{2}(x(t),u(t),t,x( \tau ), \tau ) &{} \text {for } t \ge \tau . \end{array}\right. \end{aligned}$$

Given a discount rate $\rho$, the decision maker aims at maximizing

4 $$\begin{aligned} \mathbb {E} \left[\int _{t_{0}}^{\tau } e^{- \rho t}g_{1}(x(t),u(t),t) \,\,\, \mathrm{d}t+e^{-\rho \tau }V^{*}(x( \tau ), \tau ) \,\,\, \right] \end{aligned}$$

with respect to *u*(*t*) subject to the dynamic system (2) and the intensity rate of the switch (1). The decision maker anticipates optimal behavior in the second stage,^1^ which is reflected in the optimal value of stage 2 as defined by

5 $$\begin{aligned} V^{*}(x( \tau ), \tau ):= & {} \max _{u( \cdot )} V(x( \tau ),u( \cdot ), \tau ) \nonumber \\= & {} \max _{u( \cdot )} \int _{\tau }^{\infty } e^{- \rho (t- \tau )}g_{2}(x(t),u(t),t,x( \tau ), \tau ) \mathrm{d}t. \end{aligned}$$

Here, the function $V( \cdot )$ denotes the value of stage 2 for any admissible path of the control $u( \cdot )$ on $[\tau , \infty [$. The asterisk refers to optimal/optimized values, i.e., to the value function of the stage 2 optimal control problem.

Note that the statement of the stage 1 objective function in (4) is analogous to the objective function in [11] (equation (4)). The only difference is that in [11] the decision maker faces an exogenous salvage value function at $\tau$, whereas in our case the model changes and the decision maker faces a different optimal control model.

Assuming $$\lim _{t \rightarrow \infty } \mathcal {V} (t) z_{1}(t) =0$$ with

6 $$\begin{aligned} \mathcal {V} (t)= & {} \int _{t_{0}}^{t} e^{- \rho t'}g_{1}(x(t'),u(t'),t') \mathrm{d}t' \end{aligned}$$

7 $$\begin{aligned} z_{1}(t)= & {} e^{\int _{t_{0}}^{t} - \eta (x(t'),u(t'),t') \mathrm{d}t'}, \end{aligned}$$

and considering the value of stage 2 as a function for which $V^{*}(x( \tau ), \tau )< \infty$ holds,^2^ we can apply the reformulation into a deterministic optimal control model with infinite time horizon presented in [11] and obtain8with9and with $z_{1}(t)$ being an auxiliary state variable. The interpretation is similar to a survival probability, i.e., $z_{1}(t)$ is the probability that the switch has not occurred in the interval $[t_{0},t [$. It enters the objective function (8) similar to a discount rate, reflecting the decision maker’s anticipation that a switch will occur at some point over the course of time. The value of the second stage is included with the rate $\eta (x(t),u(t),t)$ at which the switch arrives at *t* and changes the model to stage 2 with the corresponding initial conditions.

Note that in (9) we slightly abuse the notation in the sense that $V^{*}$ only depends on *x*(*t*) and *t*, although the initial condition for stage 2 is defined by evaluating $\varphi$ in the limit (from the left) of *x*(*t*) during stage 1. Here, $\varphi$ can be understood as a function that transforms the state from stage 1 to stage 2, embracing in particular the scope for a jump. Consider, e.g., a state that measures the stock of infrastructure and a natural disaster occurring at $\tau$. Then $\lim _{t^{\prime } \nearrow t} \varphi (x(t^{\prime }))$ describes the infrastructure that has not been destroyed at $\tau$.

Note that stage 2 of the above model explicitly depends on the state variable at the switching time. This can be an important feature of certain models, as is demonstrated in the example we consider in Sect. 3. Considering stage 2 alone, the dependence on $x( \tau )$ shifts the trajectories of the canonical system similar to the explicit dependence on *t* within a non-autonomous optimal problem. Even for an autonomous optimal control problem it is not possible then to derive a (single) phase diagram of the canonical system that is valid for all states and switching times.

The optimal control models (8) and (9) can be solved with classical optimal control theory (see, e.g., [25]). The problem of the second stage is straightforward, if the state variable of stage 1 is given. However, a solution of stage 1 requires the value function of stage 2 to be expressed as a function of the state and time. This is a difficult task, even numerically. Since the optimal control model is generally non-autonomous, the value function cannot be expressed as the Hamiltonian divided by the discount rate for all possible switching times (see Proposition 3.75 in [25]). Even if the optimal control model is autonomous, the phase diagram, and thus the Hamiltonian of the model, switch when the objective functional and/or the state dynamics depend on the state at the switching time, as generally they may do. In Sect. 3, we present an example that exhibits this second property.

In order to address these difficulties, we present in the next subsection a further transformation of the model, allowing its representation as a deterministic age-structured optimal control model. This has two advantages. First, the model can be solved numerically with established methods (see [7]). Second, the age-structured optimal control representation allows a simultaneous solution of both stages. The result will represent the optimal behavior for any possible switching time and, therefore, afford a broader understanding and additional insights into the solution.

### Transformation to an Age-Structured Optimal Control Model

For expositional clarity, let us first change the notation of the state and the control variable in stage 2. From now on, we use $v(t, \tau )$ ($y(t, \tau )$) for the control (state) variable at time *t* if the switch happened at $\tau$. Note that the dependence on $\tau$ is important here, as it governs the value of the control and the state. Given a switch at $\tau$, the state dynamics during stage 2 reads

10 $$\begin{aligned} \frac{\mathrm{d}y(t, \tau )}{\mathrm{d}t}= & {} f_{2}(y(t, \tau ),v(t, \tau ),t,x( \tau ), \tau ), \qquad t \ge \tau , \nonumber \\&\qquad y( \tau , \tau )= \varphi (x( \tau ), \tau ). \end{aligned}$$

Redefining the state in the second stage accordingly for every possible switching instant, i.e., $\forall \tau \ge 0$, and again abusing notation with respect to the initial condition for the state, one obtains a state variable $y( \cdot )$, which is age-structured.

*Remark on notation* The literature on age-structured optimal control models frequently denotes by (*t*, *a*) the time arguments (*t* as time, *a* as age) of the (control and state) variables. Defining $s=t-a$, this notation is equivalent to the (*t*, *s*) notation we employ, where an explicit statement of the switching time *s* provides a clearer description in our context. For instance, every characteristic line of the optimal control model is then indicated by $( \cdot ,s)$, the switching time *s* being a more direct marker.

For the transformation of the general problem, defined in (8) and (9), to an age-structured optimal control model, we first have to transform the objective function. The following lemma presents the resulting objective function, accounting for time *t* and switching time *s*, as is defined in (10).

#### Lemma 2.1

For every admissible path of the control variables *u*(*t*) and *v*(*t*, *s*) and corresponding state trajectories, the objective function (4) of the general model can be transformed into

11 $$\begin{aligned}&\mathbb {E} \left[\int _{t_{0}}^{\tau } e^{-\rho t}g_{1}(x(t),u(t),t) \,\,\, \mathrm{d}t+e^{- \rho \tau }V(x( \tau ),v( \cdot ), \tau ) \,\,\, \right]\nonumber \\= & {} \int _{t_{0}}^{\infty } e^{- \rho t} \Big [z_{1}(t)g_{1}(x(t),u(t),t) \nonumber \\&\quad + \int _{t_{0}}^{t} z_{1}(s) \eta (x(s),u(s),s)g_{2}(y(t,s),v(t,s),t,x(s),s) \,\,\, \mathrm{d}s \Big ]\,\,\, \mathrm{d}t, \end{aligned}$$

where $V(x( \tau ),v( \cdot ), \tau )$ denotes the value of stage 2 for admissible $v( \cdot ):=v(t, \tau )$ for $t \in [\tau , \infty [$ and corresponding state trajectory (see (5) for the definition).

### Proof of Lemma 2.1

Starting from the objective function (4) and its transformation into (8), we use the explicit expression for the value of stage 2, i.e.,

12 $$\begin{aligned}&\mathbb {E} \left[\int _{t_{0}}^{\tau } e^{- \rho t}g_{1}(x(t),u(t),t) \,\,\, \mathrm{d}t+e^{- \rho \tau }V(x( \tau ),v( \cdot ), \tau ) \,\,\, \right]\nonumber \\= & {} \int _{t_{0}}^{\infty } e^{- \rho t} \Big [z_{1}(t)g_{1}(x(t),u(t),t)+z_{1}(t) \eta (x(t),u(t),t)V(x(t),v( \cdot ),t) \Big ]\,\,\, \mathrm{d}t \nonumber \\= & {} \int _{t_{0}}^{\infty } e^{- \rho t} \Big [z_{1}(t)g_{1}(x(t),u(t),t) \nonumber \\&\qquad + z_{1}(t)\eta (x(t),u(t),t) \int _{t}^{\infty } e^{- \rho (s-t)}g_{2}(y(s,t),v(s,t),s,x(t),t) \,\,\, \mathrm{d}s \Big ]\,\,\, \mathrm{d}t \nonumber \\= & {} \int _{t_{0}}^{\infty }e^{- \rho t}z_{1}(t)g_{1}(x(t),u(t),t) \,\,\, \mathrm{d}t \nonumber \\&+ \int _{t_{0}}^{\infty } \int _{t}^{\infty } e^{- \rho s}z_{1}(t) \eta (x(t),u(t),t)g_{2}(y(s,t),v(s,t),s,x(t),t) \,\,\, \mathrm{d}s \,\,\, \mathrm{d}t \end{aligned}$$

Applying Fubini’s theorem, we can now change the order of integration for the second integral and obtain

13 $$\begin{aligned}&\int _{t_{0}}^{\infty } e^{- \rho t}z_{1}(t)g_{1}(x(t),u(t),t) \,\,\, \mathrm{d}t \nonumber \\&+ \int _{t_{0}}^{\infty } e^{- \rho t}\int _{t_{0}}^{t}z_{1}(s) \eta (x(s),u(s),s)g_{2}(y(t,s),v(t,s),t,x(s),s) \,\,\, \mathrm{d}s \, \mathrm{d}t. \end{aligned}$$

**Fig. 1:**
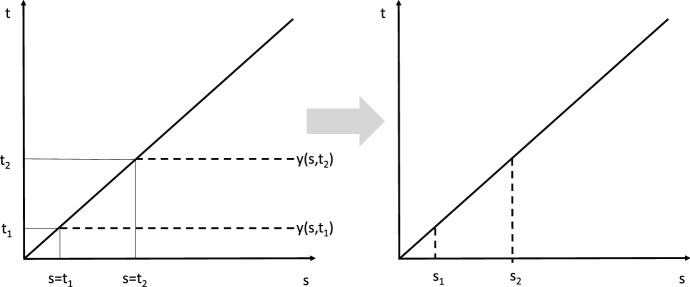
Change in the direction of summation

In contrast to the summation of the objective functional over time *s* for every switching time *t* used in the previous expression (12), we change to the summation of the objective functional over all switching times before *t*. For an illustration, see Fig. 1, where the left panel corresponds to (12): summation over time for the characteristic line starting at *t*; and where the right panel corresponds to (13): summation over all switching times before *t*. This implies that the discount factor in the second integral disappears and that age-structured optimal control theory can be applied. After rearranging terms, we arrive at (11).

The reformulation of the objective function presented in the above lemma is crucial for considering the general model as an age-structured optimal control model. To write (11) in a more compact form, we introduce the aggregate state *Q*(*t*) as sum of the objective functionals of all active characteristic lines $0 \le s \le t$ at *t*, i.e.,

14 $$\begin{aligned} Q(t)= \int _{t_{0}}^{t} z_{1}(s) \eta (x(s),u(s),s)g_{2}(y(t,s),v(t,s),t,x(s),s) \,\,\, \mathrm{d}s. \end{aligned}$$

In other words, *Q*(*t*) denotes the sum of all instantaneous objective functionals for all possible regimes (i.e., all possible switches) up to time *t*, weighted by the probability for their realization at $s\in \left[t_{0},t \right]$. Here, the instantaneous objective functionals at *t* may well depend on the state *x*(*s*) at the time of the switch. Thus, there are two time lags in the integral, which complicates the use of the standard form of the Maximum Principle. To avoid this complication, we define two auxiliary state variables $z_{2}(t,s)$ and $z_{3}(t,s)$ in the following way

$$\begin{aligned}&\frac{\mathrm{d}z_{i}(t,s)}{\mathrm{d}t} =0, \quad i=2,3, \forall t \ge s, \\&z_{2}(s,s)=z_{1}(s) \eta (x(s),u(s),s), \,\, z_{3}(s,s)=x(s). \end{aligned}$$

Here, $$z_{2}(t,s)$$ denotes the probability that the switch happened at *s*, where $$z_{2}(s,s)=z_{2}(t,s)$$$$\forall t \ge s$$ reflects that, for any switching point *s*, this probability does not change over time. Analogously, $$z_{3}(t,s)$$ denotes the value of the state variable at the switching time *s*. Using this in (14), it is possible to eliminate the time lag and write

15 $$\begin{aligned} Q(t)= \int _{t_{0}}^{t} z_{2}(t,s)g_{2}(y(t,s),v(t,s),t,z_{3}(t,s),s) \,\,\, \mathrm{d}s. \end{aligned}$$

Finally, Lemma 2.1 and the above calculations result in the following theorem.

#### Theorem 2.1

A multi-stage optimal control model with random switching time, i.e., problem (4) subject to (2), (1) and (5), is equivalent to the following age-structured optimal control model:16

This problem can be solved with age-structured optimal control theory [21–23] and established numerical methods [7].

The transformation of the multi-stage optimal control model with a random switching time ((4) subject to (2), (1) and (5)) to a deterministic optimal control model (8) enables the application of the standard Maximum Principle for a given value function (depending on the state and time), relating to stage 2 of the original problem. Thus, the stage 2 problem has to be solved first and used for the first-order conditions of the original problem [(4) with respect to (2)]. This way of deriving the optimal solution will be referred to as *backward approach*. As compared to this, working with the transformed age-structured optimal control problem (Theorem 2.1) has considerable advantages:

*Numerical solution* Applying the backward approach makes it necessary to calculate the value function of stage 2, depending on the state and on time. This is manageable (by deriving the stable trajectories of the canonical system and evaluating the slice manifold; for details we refer to [25]) if the stage 2 problem is autonomous and if neither the objective functional nor the dynamics depend on the state at the switching time, i.e., if $$g_{2}(x(t),u(t),t,x( \tau ), \tau )=g_{2}(x(t),u(t),t)$$ in (3) and if $$f_{2}(x(t),u(t),t,x( \tau ), \tau )$$$$=f_{2}(x(t),u(t),t)$$ in (2). Non-autonomy and/or dependence on the state at the switching time is likely to imply huge numerical effort, as it leads to a shift in the phase diagram. The stage 2 optimal control problem would then have to be solved for every admissible state and every *t*. In contrast, the problem is solved at a single blow in the age-structured optimal control form, as it is no longer defined over the two distinct stages. Here, established numerical methods (see [7]) can be applied.

*Analytical insights* The general model formulated in (8) and (9) includes stochasticity (i.e., a random time horizon) and two non-trivial optimization problems, one being nested in the other (i.e., the value function of stage 2 as salvage value of stage 1). The representation as an age-structured optimal control model (see (16) in Theorem 2.1) is deterministic and includes both stages simultaneously. The switching rate is naturally included as a function that depends on the control and state variables. Thus, the model, the first-order conditions and the dynamics can be presented in a compact way, allowing one to incorporate explicitly and intuitively the interaction between the two stages and the switching rate. This comes at the expense of three additional state variables, where $z_{1}(t)$ can be interpreted as a survival probability, and where $z_{i}(t,s)$ ($i=2,3$) adjust for the time lag. This complication, however, is then independent of the number of control and state variables in the original model, allowing the addition of a lot of detail without compromising the tractability of the transformed model. In contrast, the complexity of the backward solution (see previous item) strongly depends on the number of control and state variables, as the value function has to be derived for every switching time and every possible value of the state variables.

*Model illustration* The age-structured optimal control approach offers additional ways for illustrating the results of the model. In particular, it is now possible to represent the dynamics of the control and state variables across the range of switching times, i.e., $\frac{\mathrm{d}v(t,s)}{\mathrm{d}s}$, in addition to the more common dynamics over time, i.e., $\frac{\mathrm{d}v(t,s)}{\mathrm{d}t}$. Combining the two, this also allows for an easy representation of the role of duration *t*–*s*. Section 3 provides both analytical and visual representations of the dynamics for a numerical example. Altogether, the broader scope for illustrating the model dynamics is possible because in the age-structured optimal control formulation switching time is represented as an independent variable *s*, whereas the backward approach represents stage 2 by an isolated optimal control problem.

In the next section, we present a simple model of catastrophic macroeconomic disaster to illustrate the above transformation together with a numerical solution.

## Example: Preventing and Responding to Catastrophic Macroeconomic Disaster

In the light of rising concerns about catastrophic changes to environmental conditions due to climate change (see, e.g., [26]) and the reduction in biodiversity, a growing interest has emerged in the modeling of rare macroeconomic disasters (see, e.g., [4, 5, 27–29] on the modeling of catastrophic climate change and [30] for a general survey on macroeconomic disasters). The modeling of a singular catastrophic macroeconomic shock is a natural application for our framework, where in stage 1 the economy operates under the risk of a severe disaster, the arrival of which can be lowered by preventive investments; and where stage 2 is characterized by, e.g., a vastly diminished capacity for production. As is pointed out in [5], one important feature of such catastrophic shocks is that they yield permanent, or at least very long-lasting impacts.

In the following, we provide a simple, highly stylized model of such a setting, which aims at illustrating how our transformation approach works and to what uses it can be gainfully employed. Within this section, we use subscript (superscript) *i* to indicate variables (functions) for stage $i=1,2$. In the stage before a shock takes place, referred to as stage 1, we have the following setup. The economy produces output with capital stock $K_{1}(t)$ according to the production function $F^{1}(K_{1}(t))$. This output can be consumed, $c_{1}(t)$, invested to increase the capital stock in production, or invested into a protective capital stock *D*(*t*) to reduce the risk of a disaster and/or the negative impact of such a disaster in the follow-up, referred to as stage 2. Investments into protective capital are denoted by *p*(*t*). Protective capital is built up through investments according to *h*(*p*(*t*)) and depreciates at a constant rate $\delta$. The decision maker aims at maximizing the stream of utility from consumption $u(c_{1}(t))$. The shock to the economy is assumed to take place at a rate $\eta (D(t))$ that falls in the stock of protective capital. For concreteness, one could think, for instance, of $\eta (D(t))$ as a risk of permanent flooding which diminishes in the capital stock *D*(*t*) invested in the strength and height of dams and other means of flood protection.

Altogether, the model reads17where $V^{*}(D( \tau ),K_{1}( \tau ))$ denotes the value of stage 2, which is defined similarly. The difference is that physical capital is less productive in stage 2 due to the negative effect of the disaster, i.e., $F^{2}(K,D) \le F^{1}(K)$ ($K>0$, $\forall t$). The negative impact is mitigated by the protective capital at the time of the shock, i.e., $F^{2}_{D}( \cdot )>0$. Protective capital is assumed to be fixed during stage 2, implying no further depreciation and the impossibility of further investment. Altogether, the stage 2 model reads18Concavity is assumed for the utility function, the production function and the investment function into protective capital.^3^

Applying the transformation described in Theorem 2.1, the model can be reformulated as the following age-structured optimal control model19This compact representation of model (17) and (18) highlights the advantage of a transformation into an age-structured optimal control model (see ’analytical insights’ on p.17). The model is deterministic, the switching rate enters in the dynamics of $z_{1}(t)$, and both stages are considered simultaneously.

The standard Maximum Principle for age-structured optimal control theory (see [22]) can be applied to this problem, yielding first-order conditions for the controls (ensured to be positive by appropriate Inada conditions for $u( \cdot )$ and $h( \cdot )$) and corresponding adjoint equations (with suitable transversality conditions). For further details, we refer to [1].

By differentiating the first-order conditions with respect to time, *t*, and switching time, *s*, respectively, we obtain the dynamics of the control variables (their dependence on *t* and *s* as well as on control and state variables being suppressed for clarity). $\lambda _{i} (t)$ ($i=1,2,3$) denote the adjoint variables for the states $K_{1}(t)$, *D*(*t*) and $z_{1}(t)$, respectively, and $\xi _{i} (t,s)$ ($i=1,2,3$) denote the adjoint variables for $K_{2}(t,s)$, $z_{2}(t,s)$ and $z_{3}(t,s)$, respectively.

20 $$\begin{aligned} \dot{c}_{1}(t)= & {} \big ( \rho -F_{K_{1}}^{1} \big ) \frac{u_{c_{1}}}{u_{c_{1}c_{1}}} -\eta \frac{u_{c_{2}}-u_{c_{1}}}{u_{c_{1}c_{1}}} \end{aligned}$$

21 $$\begin{aligned} \dot{p}(t)= & {} \underbrace{- \frac{h_{p}}{h_{pp}}}_{>0} \Big ( \underbrace{\delta + F^{1}_{K_{1}}}_{i} - \underbrace{\frac{h_{p} \xi _{3} - \xi _{1}}{e^{-rt}z_{1}u_{c_{1}}}}_{ii} + \underbrace{\frac{\lambda _{3} - \xi _{2}}{e^{-rt}u_{c_{1}}} h_{p} \eta _{D}}_{iii} \Big ) \end{aligned}$$

22 $$\begin{aligned} \frac{\mathrm{d}c_{2}(t,s)}{\mathrm{d}t}= & {} \big ( \rho -F_{K_{2}}^{2} \big ) \frac{u_{c_{2}}}{u_{c_{2}c_{2}}} \end{aligned}$$

23 $$\begin{aligned} \frac{\mathrm{d}c_{2}(t,s)}{\mathrm{d}s}= & {} \underbrace{- \frac{u_{c_{2}}}{u_{c_{2}c_{2}}}}_{>0} \Big ( - \underbrace{\eta }_{i} + \underbrace{\frac{\eta _{D}}{\eta } \dot{D}}_{ii} - \underbrace{\frac{1}{\xi _{1}} \frac{d \xi _{1}}{ds}}_{iii} \Big ). \end{aligned}$$

Equations (21) and (23) are the consumption Euler equations relating to stages 1 and 2, respectively. While (23) is of the standard form and requires no further discussion, (21) contains an additional term related to the shock. If a disaster at time *t* leads to a collapse of production capabilities and, thus, of consumption, such that $c_{2}<c_{1}$, then the marginal utility of consumption satisfies $u_{c_{2}}>u_{c_{1}}$. In such a case, consumption is deferred (note that $u_{c_{1}c_{1}}<0$) in order to accumulate precautionary savings early on and, thereby, to soften the shock-related drop in consumption.

According to (22), protective investment increases over time (i.e., is deferred) in line with (i) its current opportunity cost (the latter being the return to productive capital); and declines over time (i.e., is advanced) with (ii) the excess value of protective capital over productive capital after the shock, and with (iii) the net value of reducing the risk of a disaster (note that $\eta _{D} <0$), with $\lambda _{3}$ being the value of prevention (equal to the value of survival in stage 1) and with $\xi _{2}$ being the value of stage 2.

According to (20), the experience of a later shock (i.e., a higher *s*) implies (i) a lower level of consumption, as more consumption has been advanced due to the risk of a shock; (ii) a lower level of consumption due to the accumulation of protective capital (the effect reverses if $\dot{D} <0$); (iii) a higher level of consumption, if the stage 2 value of productive capital is smaller for later shocks (i.e., if $\frac{d \xi _{1}}{ds} <0$), or, in other words, if more productive capital has been accumulated at a later arrival of the shock.

While the derivatives with respect to time can also be obtained by the standard backward approach, their derivation within the age-structured optimal control approach provides a compact and coherent representation of how the second stage determines stage 1 dynamics. The derivative with respect to switching time can only be obtained after applying the transformation into an age-structured model.

A numerical solution, based on the backward approach, would be extremely involved even for this simple model. This is because the dynamics of the state variable of stage 2 depend on the protective capital at the time of the shock (see ’numerical solution’ on p.17). This becomes obvious when deriving, for the specification detailed below, the steady-state capital stock as a function of switching time *s*, i.e.,

24 $$\begin{aligned} \hat{K}_{2} (s) := \lim _{t \rightarrow \infty } K_{2}(t,s)= \left[\frac{\rho }{A_{2} \beta } \left( 1-e^{- \bar{\eta } D(s)} \right) ^{-1} \right]^{\frac{1}{\beta -1}}, \quad \forall s. \end{aligned}$$

Given that the switch has happened at *s*, the optimal solution of stage 2 then follows the stable manifold leading to $\hat{K}_{2} (s)$. Notably, the value for $\hat{K}_{2} (s)$ will vary with *D*(*s*). Thus, it is not enough to derive the slice manifold for every possible switching time *s*, but one would have to derive the value function separately, depending on both *s* and *D*(*s*).

In contrast, a numerical solution can be readily obtained for the age-structured formulation. We employ the following functional specification (the dependence on *t* and *s* being suppressed)

$$\begin{aligned} \eta (D)= & {} \eta e^{- \bar{\eta } D}, \nonumber \\ h(p)= & {} p^{\alpha }, \nonumber \\ u(c_{i})= & {} c_{i}^{\sigma }, \quad i=1,2 \nonumber \\ F^{1}(K_{1})= & {} A_{1}K_{1}^{\beta }, \nonumber \\ F^{2}(K_{2},z_{3})= & {} A_{2}K_{2}^{\beta }(1-e^{- \bar{\eta } z_{3}}), \end{aligned}$$

with parameter values, $\alpha =0.75$, $\beta =0.5$, $\sigma =0.75$, $\eta =0.25$, $\bar{\eta } =0.5$, $\rho =0.03$, $\delta =0.2$, $A_{1}=A_{2}=0.75$. Furthermore, we set the initial capital stock at $K_{10}=50$.

Note that the utility function is continuously differentiable in $c_{i}$ and does not depend on any state. Thus, the assumptions concerning the objective functional (see (3) on p. 7) are fulfilled. Similarly, the production functions of both stages are continuously differentiable in the states, implying that the stated assumptions are fulfilled (see (2) on p. 7).

**Fig. 2:**
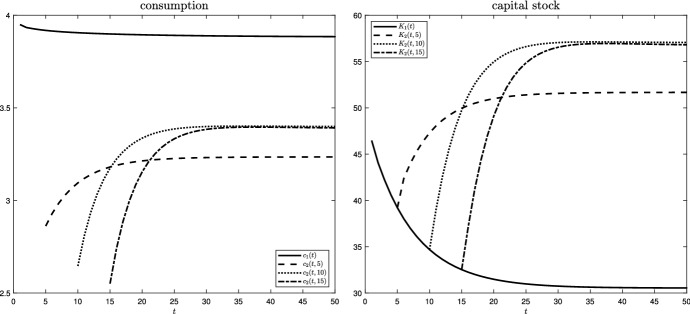
Consumption and productive capital stock over time (both stages) for $s =5, 10, 15$

**Fig. 3:**
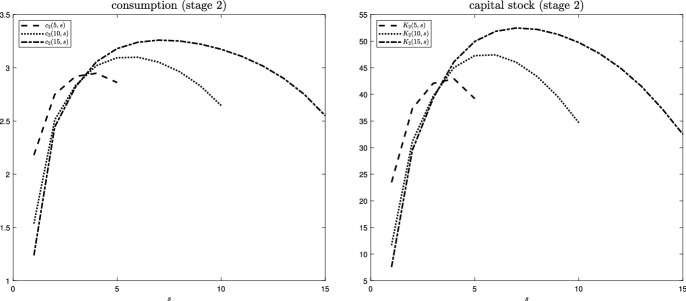
Stage 2 consumption and stage 2 productive capital stock across switching times

**Fig. 4:**
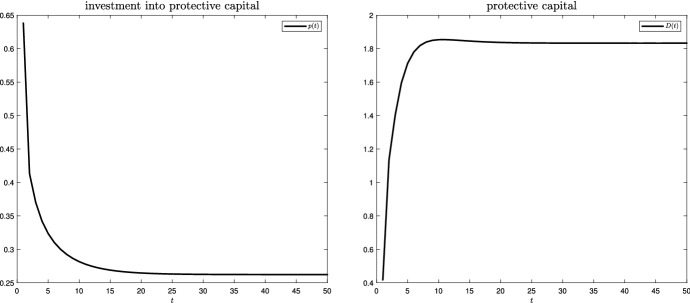
Protective investments and protective capital over time

The key outcomes are illustrated in Figs. 2, 3 and  4. The left panel of Fig. 2 plots how consumption develops over time for stages 1 and 2, depending on the arrival of the disaster at $s =5, 10, 15$. As long as no disaster hits, $c_{1}(t)$ declines and converges toward a steady state. At the point of a disaster at *s*, consumption drops sharply. Although $c_{2}(t,s)$ recovers afterward, it converges to a new steady-state level below the one of stage 1. The lower level of consumption is implied by the detrimental impact of the disaster on productivity and, thus, on total output. Similarly, the right panel of Fig. 2 plots various surfaces of the productive capital stock, *K*. During stage 1, the capital stock decreases from a high initial value toward the steady-state value it would attain in the absence of a shock. In case of a disaster, the capital stock does not drop. The production function, however, is less effective, implying that a higher steady-state capital stock needs to be built up during stage 2. The level of this steady-state capital stock then depends on the timing of the shock: early shocks, for which the impact on productivity was strong and lasting due to a low level of protective capital, inhibit even the long-run accumulation of physical capital, leading to a lower steady-state level (see (24)).

The left panel of Fig. 3 plots stage 2 consumption at time $t=5, 10, 15$ (corresponding to the three curves), depending on the time $s\le t$ at which the disaster hits. While this figure can be plotted directly after our transformation, it could only be developed under considerable effort when using the backward solution (see paragraph “model illustration” on p. 18). Increasing *s* for any given *t* implies a shorter duration since the disaster. It can be seen that, at any point in time *t*, the consumption level varies in a non-monotonous way with the duration since the shock. If disaster has just occurred (corresponding to the respective end points of the three curves), consumption is low due to the instantaneous impact. Consumption is also low (and sometimes lower) for early realizations of the shock (corresponding to the LHS end points of the three curves), where the low level of protective capital disallows a recovery of the economy. By contrast, consumption is highest for intermediate realizations of the shock, for which (a) there was sufficient time for recovery as opposed to later realizations, while at the same time (b) the recovery process was more effective than for earlier realizations. The RHS mirrors the insights from the LHS. Similar to consumption, the stage 2 level of the productive capital stock depends in a non-monotonous way on the duration since the shock. A short duration since the shock (i.e., at the end points of the three curves) implies that very little productive capital could be accumulated, starting from a low level. By contrast, a long duration since the shock implies a comparatively slow rebuilding of the capital stock due to a strong permanent decline in capital productivity for early shocks. Once again, the capital stock is highest for intermediate durations, where the time available for capital rebuilding and its effectiveness are well balanced. Recall that the time and scope for capital rebuilding also explains the stage 2 allocation of consumption.

Figure 4 plots protective investments, *p*, (left panel) and protective capital, *D*, (right panel) over time. Investments are very high at the beginning, as the steady-state level of the protective capital stock has to be built up. The protective capital stock increases until a steady state is reached.

We conclude by recalling that the numerical example lacks important modeling features, as well as the necessary calibration, that would allow one to explain the economics of real-world catastrophic disasters, such as climate shocks. With the present analysis predominantly serving as an illustration of how the transformation of a multi-stage model with random switching time into an age-structured optimal control model can be usefully applied, the formulation and analysis of a more realistic model are relegated to future work.

## Conclusions

The paper considers multi-stage optimal control models with a random switching time. Although the model can be transformed into a deterministic optimal control model following the backward approach in [11], the numerical solution remains involved. This is the case, in particular, if the objective functional or the state dynamics depend on the state, evaluated at the switching time, or on the the switching time itself. Transforming the model into an age-structured optimal control model allows one to derive the solution of both stages simultaneously. This is a considerable numerical advantage. Moreover, owing to the unified representation of both stages, the age-structured optimal control formulation offers additional analytical insights and the scope for a complete representation of the dynamics, in particular, when it comes to studying the impact of the timing of the shock and the duration since.

Naturally, the assumptions concerning the switch can be extended in various ways. In future work, we intend to allow for multiple switches, where we need to distinguish whether the switches are independent or whether they are linked through model states. Another important extension involves the modeling of a distributed impact of the switch. In our example of a natural disaster, for instance, not only the arrival of the shock is random but also its severity. The distribution of severity (for different arrival dates) would then have to be considered as an additional part to the control problem.

## Appendix A

*Innovation* While the probability of arriving at an innovation can be influenced by education (at individual or societal level) or R&D investments (at firm or government level), a technical breakthrough remains a stochastic event. Some innovations have the power to change considerably the dynamics of firms (e.g., new products; drastic innovations that lead to the domination of the market) or societies (e.g., a carbon-free backstop technology; a vaccine that leads to the eradication of certain infectious diseases, or a comprehensive anticancer treatment).

*Natural disasters/climate change* While the prevention of and response to natural (e.g., storms, flooding, volcano eruptions, earthquakes) or man-made environmental disasters (e.g., oil spills, chemical or nuclear accidents) provides a long-standing context for such analysis, the growing prospect of collapse of particular climate patterns (e.g., a standstill of the Gulf stream due to the erosion of thermal differentials within the Atlantic ocean; a substantial weakening of the jet stream; or a polar meltdown) is adding a global scale to the issue.

*Political shocks* With revolutions or landslide political change, societies can experience shock-like political events with potentially far-reaching economic and social consequences. These experiences raise issues about optimal patterns of investment in the prevention or arrival, for that matter, of radical political change. Similar issues relate to the art of “brinkmanship,” where negotiations are structured in a way that maximizes the domestic objective, while at the same time containing the risk of an international crisis.

## Footnotes

1 By assuming perfect rationality, we follow the extant literature in economics and management. Assuming biased expectations would not change our approach in qualitative terms.

2 Note that the conditions on $g_{2}( \cdot )$ and $f_{2}( \cdot )$ imply that the value function $V^{*}( \cdot )$ is continuously differentiable in *x* (see [25]).

3 Our model bears a lot similarity to an innovative approach developed by van der Ploeg and de Zeeuw (see [4, 5]) to analyze optimal policy-making in the face of a potentially catastrophic climate shock. While lacking the detail of [4, 5], approach differs (i) in not relying on the conventional backward approach to solving the model, and (ii) by including with the production function $F_{2}(K_{2}(t),D( \tau ))$ a dependency of the stage 2 problem on a state at the point of transition.
